# Intrathoracic Rib Associated with Pulmonary Collapse in a Pediatric Patient

**DOI:** 10.5812/iranjradiol.8608

**Published:** 2012-11-20

**Authors:** Fatmagul Basarslan, Hanifi Bayarogulları, Murat Tutanc, Vefik Arica, Cahide Yilmaz, Ramazan Davran

**Affiliations:** 1 Department of Pediatrics, Research Hospital, Faculty of Medicine, Mustafa Kemal University, Hatay, Turkey; 2 Department of Radiology, Research Hospital, Faculty of Medicine, Mustafa Kemal University, Hatay, Turkey

**Keywords:** Congenital Abnormalities, Tomography, Spiral Computed, Child

## Abstract

The ribs are essential structures of the osseous thorax that provide certain significant information and aid interpretation of radiologic images in daily routine practice. Intrathoracic rib is a rare congenital anomaly that is usually discovered incidentally, but may cause in vain interventions in case of being unaware. We herein report an intrathoracic rib in a girl whose chest X-ray was strange enough to obtain a spiral computed tomography (CT) scanning for a definitive diagnosis afterwards.

## 1. Introduction

Ribs are composed of three developmental compartments and embryologically originate from the somites (1). In around the fourth to sixth week of fetal life anything that damages precartilage development such as fetal intrathoracic pressure, genetic factors and chemical hazards may cause rib abnormalities (1, 2). One of these abnormalities; namely, intrathoracic rib is a rare congenital anomaly characterized usually by a supernumerary rib that runs an abnormal course in the thoracic cavity (3, 4). In this paper, we report the case of an intrathoracic rib to refresh our knowledge and to accentuate the importance and usefulness of a spiral CT. To our knowledge, this is very rare congenital anomaly which was observed for the first time in association with pulmonary collapse in the literature.

## 2. Case Presentation

**Figure 1:**
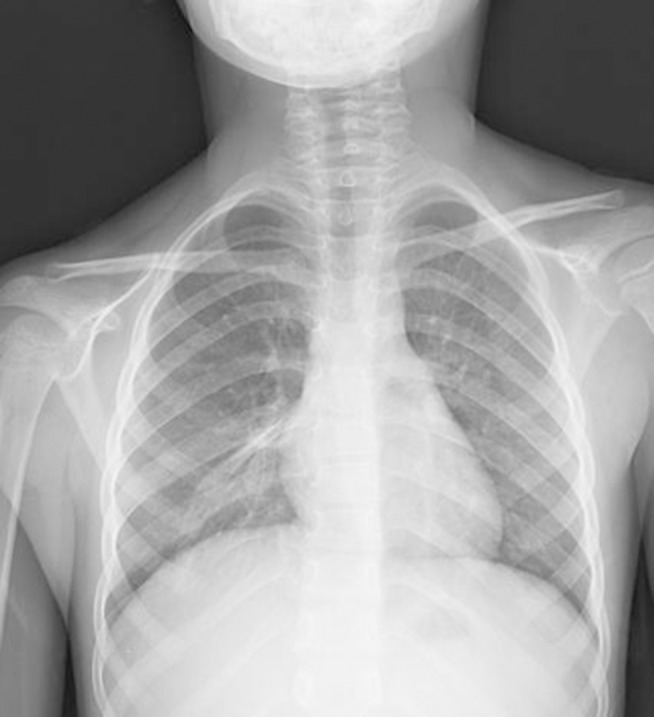
Chest X-ray; right perihilar hyperlucent appearance

**Figure 2:**
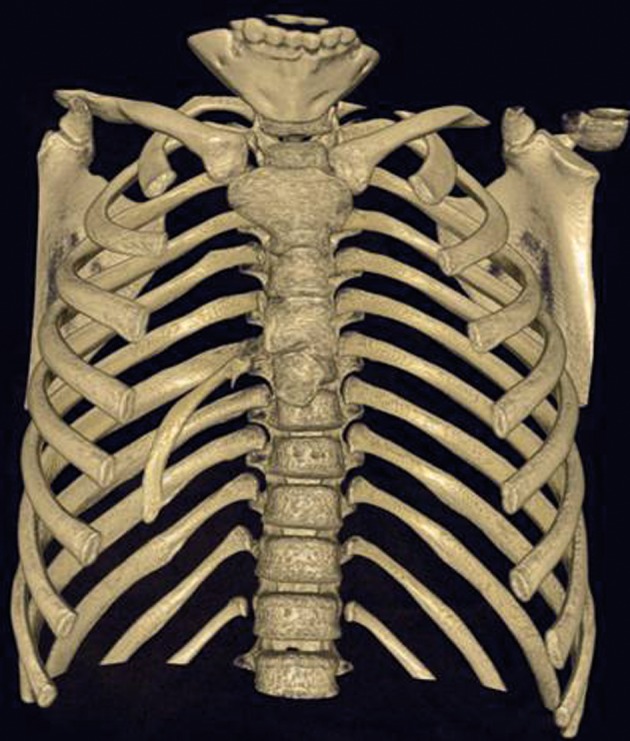
Spiral CT, anterior appearance. A right intrathoracic rib which is articulated on the anterior aspect of the T7 vertebral body and protrudes into the thorax cavity causing collapse of the lower lung parenchyma

**Figure 3:**
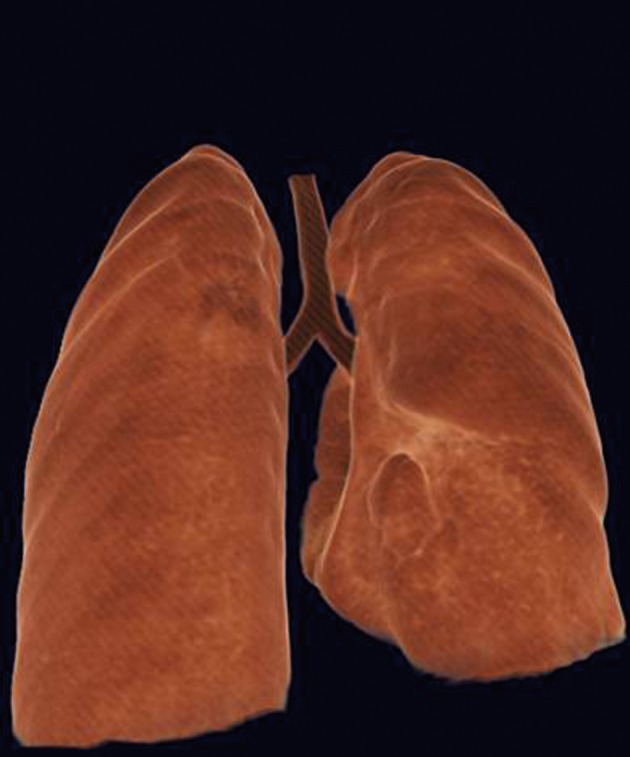
Collapse of the lower lung parenchyma on the right side

**Figure 4:**
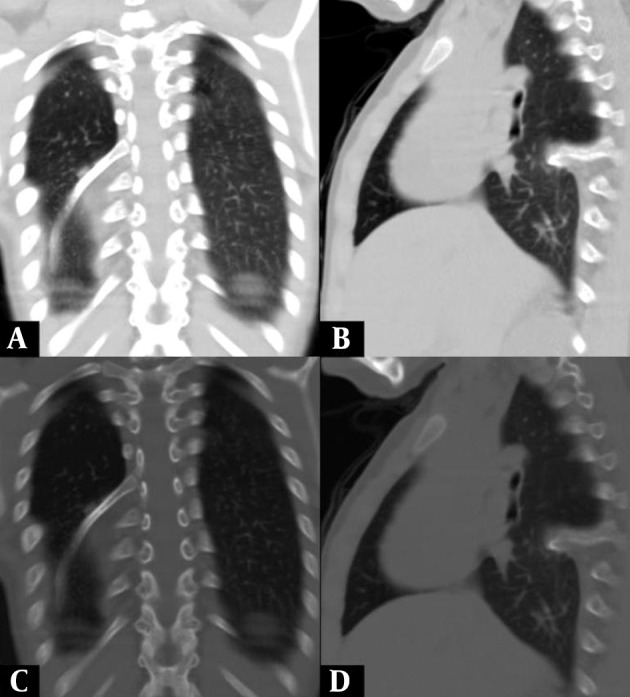
A and B, Lung window; A, Coronal plane; B, Sagittal plane; C and D, Bone window; C, Sagittal plane; D, Coronal plane In the coronal plane; intrathoracic rib that originates from the 7th thoracic vertebra and migrates inferolaterally is being observed (A, C). In the sagittal plane, an intrathoracic rib which has grown out of the corpus of the 7th thoracic vertebra and forward towards the right hemithorax space, causing pulmonary collapse at this extension. Furthermore, the lung parenchyma was posteriorly collapsed below this level to the diaphragm (B, D).

A six-year-old girl was taken to the hospital with symptoms of respiratory tract infection (respiratory distress and fever). She had no history of lung or any other systemic disease. She had also not suffered from chest trauma and surgery before, but she had scoliosis. The routine laboratory blood test results were within normal limits. A hyperdense lesion on the right hemithorax linearly extending inferolaterally from the medline was seen on chest X-ray (Figure 1). Spiral CT was obtained to clarify the situation, which revealed a right intrathoracic rib articulated on the anterior aspect of the T7 vertebral body and protruding into the thorax cavity leading to collapse of the lower lung parenchyma (Figures 2 and 3). In lung and bone window CT, in the coronal plane, an intrathoracic rib originates from the 7th thoracic vertebra and migrates inferolaterally. In the sagittal plane, it is seen that an intrathoracic rib has grown out of the corpus of 7th thoracic vertebra and forward towards the right hemithorax causing pulmonary collapse at this extension. Furthermore, the lung parenchyma was posteriorly collapsed below this level to the diaphragm (Figure 4A-D).

## 3. Discussion

There are various types of congenital anomalies of the ribs, including developmental fusion. These anomalies occur in 0.15%-0.31% of the population (5) and the rarest one is an intrathoracic rib. Approximately forty cases have been reported so far until first described in 1947 (6). The development of this anomaly may be explained by both certain alterations in gene expressions and incomplete fusion of the sclerotome, from which the rib originates normally (1, 2). They are more commonly asymptomatic, unilateral and on the right side with no gender predilection (2). Approximately one-third of the reported cases are in children. It is usually found incidentally in both the pediatric and adult population and causes misdiagnosis in the majority. The case was also on the right side and was detected incidentally. It did not result in any complaint at all. The first classification of intrathoracic ribs represented in 2006 (7). Type I-a is an intrathoracic rib originating from a vertebral body and type I-b is an intrathoracic rib originating from a rib.

Type II is described as a bifid intrathoracic rib originating from a distal rib. Type III is a rib depressed into the thoracic cavity. The fourth type represents combination of type II and type III. This case resulted from the vertebral body, therefore it was considered as a type I-a.

An intrathoracic rib is an innocuous congenital anomaly which usually does not require any treatment. Although no treatment was applied in the case, it may bring about an uncertainty in diagnosis and an unnecessary therapy. So spiral CT scan has an important role in highlighting the circumstances. In our patient, it accurately characterized the intrathoracic rib with its appearance and origin (Figures 2 and 3). Similarly, in the present case, an intrathoracic rib and partial parenchymal collapse on the the right lung were clearly detected by spiral CT. To our knowledge, there have been approximately 40 cases of intrathoracic rib reported in the literature till now. So we found it expedient to report the case in order to contribute to the literature due to very demonstrative spiral CT images.

In conclusion, intrathoracic ribs should be kept in mind in the differential diagnosis of parenchymal lesions. The diagnosis without any necessary intervention is essential for contemporary medicine at present. Spiral CT demonstrates the origin and extent of the intrathoracic rib with high accuracy. It therefore appears to be the modality of choice for this very rare anomaly as well.
